# The financial burden of cancer: Estimates from patients undergoing cancer care in a tertiary care hospital

**DOI:** 10.1186/1475-9276-11-60

**Published:** 2012-10-15

**Authors:** Adnan A Zaidi, Tayyaba Z Ansari, Aziz Khan

**Affiliations:** 1 Shaukat Khanum Cancer Memorial Hospital, Main Clifton Road, Clifton, Karachi, Pakistan; 2 Aga Khan University Hospital, Stadium Road, P.O. Box 3500, Karachi 74800, Pakistan

**Keywords:** Cancer, Financial burden, Health care, Support program

## Abstract

**Introduction:**

The emotional burden associated with the diagnosis of cancer is sometimes overshadowed by financial burden sustained by patient and the family. This is especially relevant for a developing country as there is limited state support for cancer treatment. We conducted this study to estimate the cost of cancer care for two major types of cancer and to assess the perception of patients and families regarding the burden of the cost for undergoing cancer treatment at a private tertiary care hospital.

**Methods:**

This cross-sectional study was conducted at day care and radiotherapy unit of Aga Khan University, Hospital (AKUH) Karachi, Pakistan. All adult patients with breast and head & neck cancers diagnosed for 3 months or more were included. Data was collected using a structured questionnaire and analysed using SPSS.

**Results:**

Sixty seven patients were interviewed during the study period. The mean and median monthly income of these patients was 996.4 USD and 562.5 USD respectively. Comparatively the mean and median monthly cost of cancer care was 1093.13 USD and 946.42 USD respectively. The cost of the treatment either fully or partially was borne by the family in most cases (94%). The financial burden of cancer was perceived as significant by 28 (42%) patients and unmanageable by 18 (27%) patients. This perceived level of burden was associated significantly with average monthly income (*p* = <0.001).

**Conclusion:**

Our study indicates that the financial burden of cancer care is substantial and can be overwhelming. There is a desperate need for treatment support programs either by the government or other welfare organisations to support individuals and families who are already facing a difficult and challenging situation.

## Introduction

The diagnosis of cancer is shocking news for any individual and his/or her family. Cancer is a chronic disease and the physical and emotional burden can sometimes be overshadowed by the financial burden incurred by the family. In developed countries and more affluent societies of the world, a large part of this burden is shared by the state. But even so, studies from the developed countries like Canada [1] have shown that patients and families experience significant burden despite several state plans that are in place to address the issue. Wage losses due to cancer treatment in working women with breast cancer adversely affect the barely manageable situation and add to the financial burden sustained by the patient [2]. Another study from the USA showed that financial burden can be substantial even among women with comprehensive health insurance policies [3]. Similarly, a study from Australia publicised that lost income, health service expenditures and lost unpaid work were the greatest sources of economic burden among women with breast cancer [4].

In Pakistan, like many other developing countries, there is little assistance from the government and in many cases the entire costs, including direct and indirect costs, are borne by the patient and his or her family. To our knowledge there has been no study conducted in Pakistan to address this important issue. There is also very limited data from other developing countries facing a similar situation. Therefore, we conducted this study to estimate the cost of cancer care for two major types of cancers at Aga Khan University Hospital (AKUH). This is a private university hospital in Karachi that offers comprehensive cancer care. We also assessed the perceived financial burden of cancer care among patients and their care givers.

## Methods

This cross-sectional study was conducted at Aga Khan University, Hospital (AKUH) Karachi Pakistan from March 2009 to March 2010. All adult patients who had been diagnosed with either breast or head & neck cancers for at least three months were included in the study. Patients were enrolled from the Day-care chemotherapy and radiation therapy units. Interviews were conducted from the patients and/or family members and data was collected using a structured pre-tested questionnaire. Interviews were conducted by a medical student after initial pilot testing. Questions included demographics, family income, treatment costs, insight regarding the treatment and expectations of the patients and families. Complete confidentiality of the information collected was ensured. No personal data was collected by which the participants could be identified afterwards. Written consent was taken from all participants. The study was approved by the Aga Khan University’s Ethical Review committee.

### Statistical analysis

Data was analysed using commercially available software package for social science SPSS version 17. All costs were estimated as Rupees per month and later converted into dollars for analysis. The conversion rate of 2009-2010 was used and applied to other studies for comparison. Descriptive analysis was carried out for patients’ demographics and clinical characteristics. Means with standard deviations were calcula*t*ed for continuous variables and proportions were calculated for categorical variables. Chi-square test was used for Univariate analysis for significance of categorical variables in determining perceived level of burden and cost anticipation of the treatment. Logistic regression was done for same categorical variables in multivariate analysis. The significance level was set at 5%. Student’s t-test was applied for multiple values of test variables.

## Results

In all, 67 patients were interviewed. Of these 44 (66%) were females and 23 (34%) were males. The mean age of males and females was 42.6 and 46.8 years respectively. The majority of the responders were from Karachi (79%). The distribution of the type of cancer shows marked variation between genders as most females had breast cancer (91%) and all males had head and neck cancer (100%). Overall 66 patients gave information about their total monthly income. The mean and median monthly family income of these patients was 996.4 USD and 562.5 USD respectively. Comparatively, the mean and median monthly cost of cancer care was 1093.13 USD and 946.42 USD respectively. The patient was the primary bread earner in 38.8% of the cases. The characteristics of the patients are shown in Table 1.

**Table 1:** Characteristics of patients undergoing breast and head and neck cancer care at Aga Khan University Hospital

**Characteristics**	**Frequency (% age)**	**Gender**	
		**Male (n=23)**	**Female (n=44)**
**Age Groups**			
25-45 years		12	22
≥46-60 years		11	22
**Mean age(years)**		42.6	46.8
**Residence**			
Karachi	53 (79)	16	37
Outside Karachi	14 (21)	7	7
**Type of cancer**			
Breast	40 (59.7 )	0	40
Head and Neck	27 (40.3 )	23	4
**Patient bread earner before diagnosis**			
Yes	26 (38.8 )	21	5
No	41 (61.2 )	2	39
**Cost borne by patient or family**			
Completely/Partially	63 (94 )	21	42
Third party support	4 (6 )	2	2

The overall average duration of the treatment was 6.7 months, that for breast cancer was 7.8 months and for head and neck cancer was 5.04 months. The cost of the treatment was either fully or partially borne by the patient or the family in 94% of the cases.

Regarding cost anticipation, 20 (29.9%) patients confirmed that the costs were ‘more’ than anticipated or presumed, 29 (43.3%) responded that they were ‘much more’ than anticipated while for 18 (26.9%) patients the costs were ‘not’ more than anticipated. Less than half (44.8%) of the total patients (30 out of 67) informed that they were aware of the cost at the start of the treatment. The association between cost awareness and cost anticipation was statistically significant (*p*=0.01). The univariate and multivariate analyses for cost anticipation is shown in Table 2.

**Table 2:** Univariate and multivariate Analysis for Cost anticipation

**Variables**	**Cost more than anticipated**	**Cost not more than anticipated**	**Univariate analysis**	**Multivariate analysis**
	**n (%)**	**n (%)**	***p***	***p***
Gender				
Male	19(83)	4(17)	0.26	0.43
Female	30(68)	14(32)		
Age Groups			0.41	0.43
25-45 years	23(68)	11(32)		
≥46-60 years	26(79)	7(21)		
Awareness of cost at outset				
Yes	17(57)	13(43)	0.01	0.03
No	32(86)	5(14)		
Type of cancer				
Breast	28(70)	12(30)	0.58	0.62
Head and neck	21(78)	6(22)		
Current residence				
Karachi	39(74)	14(26)	1.0	0.79
Outside Karachi	10(71)	4(29)		
Income groups				
<20,000	18(86)	3(14)	0.10	0.32
21,000-50,000	16(76)	5(24)		
>50,000	14(58)	10(42)		
Total cost groups				
< 60,000	17(74)	6(26)	0.95	0.35
60,000-100000	17(71)	7(29)		
>100,000	15(75)	5(25)		

The burden of cancer was perceived as significant by 28 (42%) patients and unmanageable by 18 (27%) patients. Those who had monthly income less than 250 USD were more likely to perceive the burden as significant or unmanageable (95%) as compared to those who had monthly income more than 625 USD (37.5%) i.e. 95% of the people who had monthly income less than 250 dollars (20 out of 21) perceive the cost of the treatment as significant or unimaginable as opposed to 37.5% of responders (9 out of 24) who have monthly income more than 625 USD. The details of the income groups and their perceived level of burden are shown in Table 3.

**Table 3:** Perceived level of financial burden by income among patients undergoing breast and head and neck cancer care at Aga Khan University Hospital

**Monthly Income** **(USD)**	**Perceived level of Burden (n)**					
	**None**	**Slight**	**Some-what**	**Significant**	**Un-manageable**	**% age of perceived burden as significant & unmanageable in each income group**
<=250	1	0	0	7	13	95.2%
251-625	2	2	0	12	5	80.9%
>625	6	5	4	9	0	37.5%
Missing*		1				0.0%
Total	9	8	4	28	18	68.6%

* One patient did not give information about monthly income.

The association was not statistically significant between perceived level of burden and the type of cancer (*p*=0.79), gender (*p*=0.26), average monthly cost of treatment (*p*=0.10) or the area of residence (*p*=0.75). However, the association was statistically significant with the monthly income (*p*=<0.001). Also in multivariate analysis the monthly income was the only variable that showed significant association with the perceived level of burden (*p*=0.009). The monthly cost of the treatment barely approached significance level (*p*=0.09). The detailed univariate and multivariate analysis for perceived level of burden is shown in Table 4.

**Table 4:** Univariate and multivariate Analysis for Perceived level of burden

**Variables**	**Perceived level of burden**		**Univariate analysis**	**Multivariate analysis**
	**None to somewhat**	**Significant to unmanageable**		
	**n (%)**	**n (%)**	***p***	***p***
Gender				
Male	5(22)	18(78)	0.26	0.99
Female	16(36)	28(64)		
Age Groups				
25-45 years	12(35)	22(65)	0.60	0.15
≥46-60 years	9(27)	24(73)		
Awareness of cost at outset				
Yes	11(37)	19(63)	0.44	0.64
No	10(27)	27(73)		
Type of cancer				
Breast	12(30)	28(70)	0.79	0.99
Head and neck	9(33)	18(67)		
Current residence				
Karachi	16(30)	37(70)	0.75	0.33
Outside Karachi	5(36)	9(64)		
Income groups				
<20,000	1(5)	20(95)	0.001	0.009
21,000-50,000	4(19)	17(79)		
>50,000	15(63)	9(37)		
Monthly cost groups				
< 60,000	4(17)	19(83)	0.10	0.09
60,000-100000	9(38)	15(62)		
>100,000	8(40)	12(60)		

The monthly income and the monthly cost of treatment showed a trend towards association but that was not statistically significant (*p*=0.072). Those who had monthly income less than 250 USD were more likely to have monthly cost of treatment less than 750 USD (52.38%) as compared to those who had monthly income more than 625 USD (16.67%) as shown in Table 5.

**Table 5:** Monthly cost of cancer care by income (p=0.072) and months into treatment (p=0.116) among patients undergoing breast and head and neck cancer care at AKUH

	**Monthly cost in USD**				
	**<750**	**750-1250**	**>1250**	**Mean (±SD)**	**Total (n)**
**Monthly income (USD)**					
<=250	11	6	4	895.53 (±518.50)	21
251-625	8	9	4	960.86 (±374.76)	21
>625	4	9	11	1344.50 (±579.21)	24
Missing*			1		1
Total	23	24	20		67
**Months into treatment**					
3-5	6	10	13	1263.76 (±514.61)	29
6-8	12	9	6	1005.94 (±563.74)	27
>9	5	5	1	857.31 (±454.58)	11
Total	23	24	20	1093.13 (±542.24)	67

* One patient did not give information about monthly income.

The association between monthly cost of treatment and months into treatment was not significant (*p*=0.116), although the mean monthly cost of treatment was much higher for those who were less than 6 months into treatment as compared to those who were more than 9 months into treatment (Table 5).

Percentages of different of types of costs are shown in Table 6. Hospitalization, surgery and investigations accounted for most of the costs with mean and median percentage of 43.40 and 42.97 respectively. Doctor’s fee accounted for a mean of 7.48% and median of 5.76% of the total cost of treatment. The cost of chemotherapy and radiotherapy were quite variable and could be as high as 78.33% and 45.71% respectively in some cases. The total costs were underestimated in our study by patients on account of on-going treatments.

**Table 6:** Percent of different types of costs

**Type of Expenses**	**Mean With (±SD)**	**Median**
Doctor fee	7.48 (±4.83)	5.76
Hospitalization, surgery and investigations	43.40 (±17.53)	42.97
Chemotherapy	32.82 (±18.04)	32.00
Radiotherapy	21.41 (±11.17)	20.08
Other	8.88 (±8.01)	5.92

## Discussion

Though our study targeted a selected patients’ group from a single centre, yet it points out that the stigma of the financial burden of the cancer care could be substantial and overwhelming. The financial problems and social complexities could multiply the stress and the sufferings associated with the disease itself and the toxicities of the treatment manifolds.

The financial aspect of the disease is particularly sensitive in countries like Pakistan where almost entire cost of the treatment is borne by the patient and the immediate family with little or no support from state or health insurance policies. Hence, the diagnosis of the cancer could be devastating news not only because of nature of the disease but also because of the continuous financial drain posed by the costs of the treatment.

According to IMF as of 2010 [5], Pakistan’s Gross domestic product (GDP) per capita stands at 1,067.971 US dollars while Gross domestic product (GDP) based on purchasing power parity (PPP) per capita stands at 2,713.272 US dollars. The average family income is 226.10USD/month but Pakistan is a country of extremes. According to human development report 2008 [6], 60.3% of Pakistani population has daily income of 2 US dollars or less. However, our study group represented a relatively more affluent section of the society with a median family income of 996.4 USD per month. Despite this, the monthly cost of treatment i.e.1093.13 USD, far exceeded the average monthly income of the entire ‘affluent’ family (996.4 USD). This could mean utilisation of savings and other means to bridge the deficit. In the worst scenario, this might lead to falling in debt which had been reported by 34.3% of the patients. It could be inferred from the above fact that some people from underprivileged strata of the community might succumbed to the disease without getting treatment due to the affordability issue. Nevertheless, this needs to be documented from public sector before drawing a conclusion from the assumption.

Majority of the patients in our study (73%) reported that the costs were either more or much more than anticipated and 55.2% of the study subjects stated that they were not aware of the cost of treatment at outset. The cost of the treatment was underestimated by mostly those people who were oblivious of the cost from the outset. This was again not surprising as a cost related to cancer care is not a one-time expense in most cases. This is different from a one-time expense like knee replacement etc.

In Pakistan, families still rely overwhelmingly on an income of single earning member. This is usually the male member of the house. In our study all the patients with breast cancers were females and majority of them were homemakers with little income of their own. They were completely dependent on their families for the treatment expenses. The situation was even worse for the male patients, as they were the bread earners for their families in 91% of the cases. It gets almost impossible to sustain that supporting position when cancer treatment is underway for the main earning member. The emotional and physical toll could be even more difficult when the prognosis is poor.

This is in contrast to the findings in the western countries where the hospital related costs are mostly covered by the state or insurance policies. The financial burden in the west is mostly associated with lost income etc. Therefore, studies from that part of the world usually address the non-treatment related financial burden. A study conducted in USA in privately insured women with breast cancer reported average monthly financial burden of $1, 455 [3] but the GDP of USA based on purchasing power parity (PPP) per capita is 47,701.80 USD [7]. Nevertheless,, a study carried out in USA showed that despite comprehensive health insurance policies financial burden of breast cancer can be substantial [3]. Another study carried out in Canada showed that despite the fact that treatment and hospital related costs are paid by the public health care system, patients with breast cancer face financial hardship and numerous other costs posing a significant financial burden. The costs are especially worrisome when the financial means to cover them are inadequate [8]. It would be difficult to imagine the perceived financial burden if the entire treatment costs are shifted onto the patient and family as is the case in Pakistan. The word significant and substantial has been used in these studies from the literature but percentage reporting this has been described in a different format which limited the direct comparison between developing and developed countries. Also most of the studies have addressed the out of pocket and indirect cost from patients’ perspective. The cost figures from a similar study have been shown in Table 7. We could appreciate the comparable figures of mean cost of the treatment between our study and study by Azorullah et al [3] but there is a huge variation in the monthly income which could multiply the perceived level of burden by many folds.

**Table 7:** Comparison of the overall cost from an international Study

**Studies**	**Monthly income USD**	**Monthly Cost Mean USD (SD)**	**Cost from time since treatment Mean USD (SD)**		
			**<6months**	**6-12 months**	**>12 months**
Our study	< 250 (32%)	1028 (510)	1189 (484)	920 (500)	846 (540)
	251-625 (32%)				
	>625 (36%)				
Arozullah et al [3]	< 2500 (19%)	1455 (2366)	1375 (2157)	2331 (3424)	669 (634)
	2500-5000 (27%)				
	>5000 (54%)				

In most low income countries, people have to rely on themselves and do not necessarily look out to the government for health care needs.

The cost of the treatment is of prime importance while making treatment decisions and sometimes governs the choice of the treatment selected for a particular patient. This was reflected in an interesting trend in our study where patients with low monthly income had less monthly cost of treatment as compared to those with high monthly income. Although this did not reach statistical significance, but physicians working in Pakistan could appreciate this fact where many times a treatment is tailored to the financial situation of the patient. Similar finding has been reported from a study in USA, which showed that families with the lowest household income had least expenses. However the proportion of household income spent on cancer care was higher for low income families [3].

The small sample size was a major limitation in our study. Many patients refused to consent for disclosure of details regarding the income due to sensitivity of the issue. Day care and radiotherapy unit of AKUH render its’ services to approximately 20 million population of Karachi city [9] and also receive referrals from all over the country. The patient group is diverse comprising of all ages with different malignant disorders i.e. solid tumours and lymphomas. We had targeted select types of cancers, thus, done a purposive sampling for the sake of simplicity as this was the first study of its kind from Pakistan. The age and gender distribution was a bit skewed for the very same reason. This has limited the generalisation of the results, but this could be considered a pilot study with more comprehensive studies to follow. Nevertheless, it has given an insight regarding the under-addressed prevailing issue.

## Conclusion

Our study indicated that the financial burden of cancer care was substantial and mostly borne by the patient or the family. Most of the time, the monthly average cost of the treatment far exceeded the monthly household income and a significant proportion of patients perceived the financial burden as overwhelming. There should be financial support programs on part of the government and other organisations to cover up for the treatment costs of the cancer and to help these patients in managing the already difficult and challenging situation.

## Competing interests

The authors declare that they have no competing interests.

## Authors’ contributions

AAZ conceived of the study, conducted the literature search, designed the study and formulated the questionnaire and drafted the main manuscript. AK collected and entered the data and did the initial analysis. TZA did the thorough analysis, applied statistical tests of significance, edited and reviewed the entire manuscript. TZA also responded to the reviewer’s comments and maintained the correspondence. All authors read and approved the final manuscript.

## Authors’ information

Dr Adnan Ali Zaidi

Diplomate American Board of Medical Oncology

FRCP (Canada)

Primary Author

Consultant Oncologist

Shaukat Khanum Cancer Memorial Hospital

Karachi

Dr Tayyaba Zehra Ansari MBBS; FCPS; MRCP (UK); FCPS (Oncology)

Corresponding Author

Consultant Medical Oncologist

Aga Khan University Hospital, Karachi

Stadium Road P.O.Box 3500, Karachi 74800

Pakistan
